# Clinicopathological characteristics and prognosis in patients with monoclonal gammopathy and renal damage in central China: a multicenter retrospective cohort study

**DOI:** 10.1038/s41598-024-58467-z

**Published:** 2024-04-01

**Authors:** Huimin He, Zheng Wang, Jiayun Xu, Yun Liu, Yeqing Shao, Yulong Hou, Jinping Gu, Ruimin Hu, Guolan Xing

**Affiliations:** 1 Department of Nephrology, The First Affiliated Hospital of Zhengzhou University, Zhengzhou, Henan China; 2 Department of Nephrology, The First Affiliated Hospital of Henan University of Science and Technology, Luoyang, Henan China; 3 Department of Nephrology, The First Affiliated Hospital of Xinxiang Medical University, Xinxiang, Henan China

**Keywords:** Clinicopathological characteristics, Prognosis, Monoclonal gammopathy, Renal damage, Medical research, Nephrology

## Abstract

Renal involvement is common in monoclonal gammopathy (MG); however, the same patient may have both MG and non-paraprotein-associated renal damage. Accordingly, distinguishing the cause of renal damage is necessary because of the different clinical characteristics and associated treatments. In this multicenter retrospective cohort study, we described the clinicopathological characteristics and prognosis of 703 patients with MG and renal damage in central China. Patients were classified as having MG of renal significance (MGRS), MG of undetermined significance (MGUS), or hematological malignancy. 260 (36.98%), 259 (36.84%), and 184 (26.17%) had MGRS, MGUS, and hematological malignancies, respectively. Amyloidosis was the leading pattern of MGRS (74.23%), followed by thrombotic microangiopathy (8.85%) and monoclonal immunoglobulin deposition disease (8.46%). Membranous nephropathy was the leading diagnosis of MGUS (39.38%). Renal pathological findings of patients with hematological malignancies included paraprotein-associated lesions (84.78%) and non-paraprotein-associated lesions (15.22%). The presence of nephrotic syndrome and an abnormal free light chain (FLC) ratio were independently associated with MGRS. The overall survival was better in patients with MGUS than in those with MGRS or hematological malignancies.

## Introduction

Monoclonal gammopathy (MG) is defined as the presence of a monoclonal immunoglobulin (MIg) or its components in the serum as a result of clonal multiplication of plasma cells or B lymphocytes^1,2^. MGs are a set of disorders that include benign conditions, such as MG of undetermined significance (MGUS), and malignancies, such as Waldenström macroglobulinemia (WM) and multiple myeloma (MM)^3^. The concept of MG of renal significance (MGRS) was first coined by the International Kidney and Monoclonal Gammopathy (IKMG) Research Group in 2012^4^. It is defined as the presence of kidney injury secondary to MIg and without hematological malignancy. The definition of MGRS was revised by the IKMG Research Group in 2019^5^. The new definition of MGRS refers to the presence of one or more renal lesions associated with MIg; however, the underlying plasma cell or B-cell clone does not produce tumor disorders or meet any present hematological criteria for specific treatment.

The kidney is a favorable target for MG^6^; however, the same patient may have both MG and unrelated renal injury. Accordingly, it is necessary to distinguish the cause of renal injury because of the different clinical characteristics and associated treatments^7^. The renal biopsy is the gold standard for diagnosis.

Central China has a high prevalence of renal disease in China; however, large-scale clinical studies of patients with MG and renal damage in this region are lacking. In this multicenter retrospective study, we describe the clinicopathological characteristics and prognosis of 703 patients with MG and renal damage in central China.

## Study design and methods

### Participants

This retrospective multicenter cohort study was conducted at three hospitals in central China (the First Affiliated Hospital of Zhengzhou University, the First Affiliated Hospital of Henan University of Science and Technology, and the First Affiliated Hospital of Xinxiang Medical University). All patients were selected from the three centers between January 2012 and December 2021. The inclusion criteria for the study were as follows: (1) underwent at least one renal biopsy; (2) had at least one positive result on serum protein electrophoresis (SPE), serum immunofixation electrophoresis (IFE), urine IFE, urine Bence Jones protein (BJP) electrophoresis, serum free light chain (FLC) ratio, or urine FLC ratio; and (3) had complete clinical and pathological data. Patients who met the inclusion criteria were classified as having MGRS, MGUS, or hematological malignancy. The details for patient selection are shown in Supplementary Fig. S1. The study protocol was approved by the Ethics Committee of the First Affiliated Hospital of Zhengzhou University. All methods were carried out in accordance with relevant guidelines and regulations. Informed consent was obtained from all participants.

### Clinical and laboratory assessment

Clinical and laboratory data were collected at the time of renal biopsy, including age, gender, history of hypertension, proteinuria, hematuria, serum albumin, hemoglobin, serum creatinine, estimated glomerular filtration rate (eGFR), serum complement 3 (C3), complement 4 (C4), MIg, serum FLC level, and bone marrow biopsy. The following clinical definitions were used: (1) nephrotic syndrome was defined as 24-h urine protein excretion > 3.5 g, serum albumin level < 30 g/L, with or without renal function impairment; (2) renal insufficiency was defined as eGFR < 60 mL/min/1.73 m^2^ using the Chronic Kidney Disease Epidemiology Collaboration 2009 formula; (3) abnormal FLC ratio was identified if patients with eGFR ≥ 60 mL/min/1.73 m^2^ had a ratio outside the range of 0.26–1.65 or patients with an eGFR < 60 mL/min/1.73 m^2^ had a ratio outside the range of 0.37–3.10^8^; (4) lesions were considered to be associated with MGRS based on the IKMG guidelines^5^; (5) thrombotic microangiopathy (TMA) related with MG was considered without other obvious cause for the TMA presenting (such as atypical hemolytic uremic syndrome, thrombotic thrombocytopenic purpura, drugs, or underlying autoimmune disease)^9,10^; and (6) the criteria for the diagnosis of MM and MGUS were based on the International Myeloma Working Group (IMWG) guidelines^11^.

### Renal histopathology assessment

Renal biopsies were examined by light microscopy (LM), immunofluorescence (IF), and electron microscopy (EM). Renal biopsy specimens were fixed in 4% buffered formaldehyde for LM. Serial 2 μm sections were used for histological staining, including hematoxylin–eosin, periodic acid-Schiff, periodic acid-silver, Congo red staining, and Masson trichrome. IF staining on frozen tissue included IgG, IgA, IgM, C3, C4, C1q, fibrinogen, and both κ and λ light chains. IgG subclass staining was performed in patients with positive IgG results. IF or immunohistochemical staining of paraffin-embedded tissues after enzyme digestion, immunoelectronmicroscopy, and mass spectrometry were performed if necessary. The pathological diagnoses of all cases were established by a consensus between two experienced nephropathologists.

### Follow-up

The follow-up duration was defined as the time from renal biopsy to the last follow-up visit (September 2023). Study outcomes included all-cause death and kidney disease progression. Kidney disease progression was defined as progression to end-stage renal disease (sustained eGFR < 15 mL/min/1.73 m^2^ or the need for maintenance renal replacement therapy) or a permanent 30% reduction in the eGFR relative to the initial level at biopsy.

### Statistical analyses

SPSS 20.0 statistical software was used for statistical analysis. Graphpad Prism 9.0.0 software was used to plot the survival curves. The Kolmogorov–Smirnov test was used to test the normality of continuous variables. Continuous variables were expressed as mean ± standard deviation (SD) if normally distributed and median (interquartile range [IQR]) if not. Categorical variables were presented as number and percentage. The analysis of variance (ANOVA) was used to compare continuous variables that were approximately normally distributed. The Kruskal–Wallis test was used for skewed continuous variables. The chi-squared test or Fisher’s exact test was used to compare categorical variables. We used multivariable logistic regression to analyze the odds ratio (OR) and 95% confidence intervals (CI) for developing MGRS. Survival analysis was performed by generating Kaplan–Meier curves using the log-rank test. Only patients who were followed up for ≥ 3 months were included in the survival analysis. All *P* values were two-sided. *P* < 0.05 was considered statistically significant.

## Results

### Demographic of the study subjects

**Figure 1:**
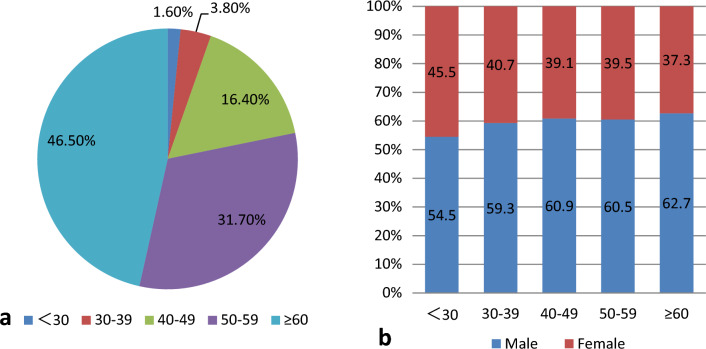
The distribution of patients with MG and renal damage based on different age groups and genders. (**a**) The distribution of patients with MG and renal damage based on different age groups. (**b**) The gender distributions of patients with MG and renal damage based on age.

In total, 703 patients with MG who underwent a renal biopsy were included in this study. The average age of the patients at renal biopsy was 58 (51, 66) years, with a male (432 cases, 61.5%) to female (271 cases, 38.5%) ratio of 1.59:1. Adolescent patients (aged < 40 years) accounted for 5.4% (38 cases) of the full cohort, with a male (22 cases, 57.89%) to female (16 cases, 42.11%) ratio of 1.375:1. Middle-aged and senile patients (≥ 40 years old) accounted for 94.6% (665 cases) of all patients, with a male (410 cases, 61.65%) to female (255 cases, 38.35%) ratio of 1.61:1. (Fig. 1a, b).

### Renal pathology findings of patients with MG and renal damage

**Figure 2:**
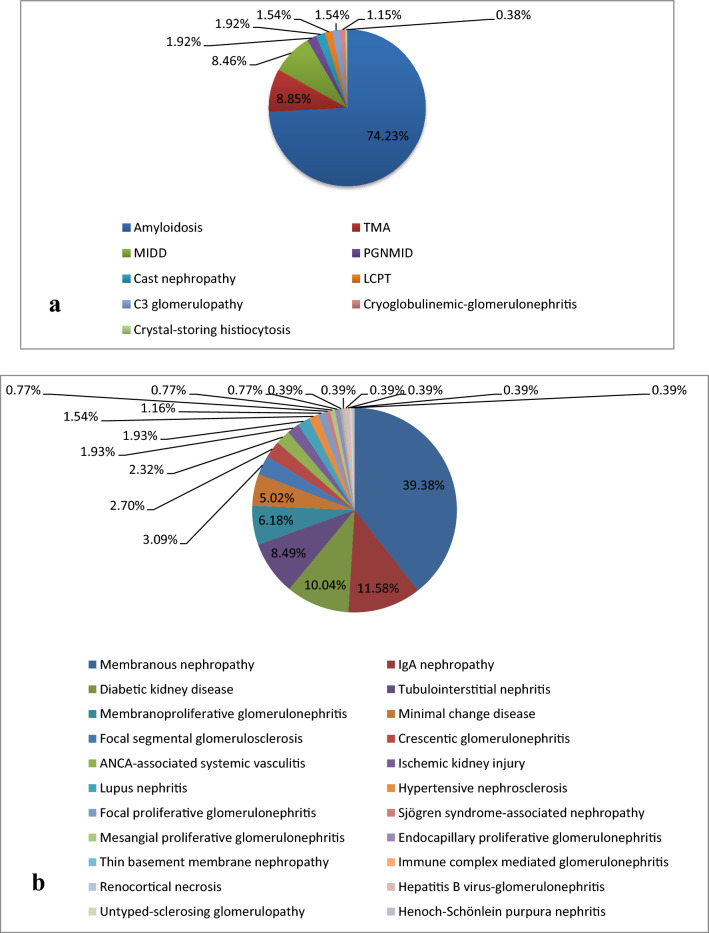
The renal pathology of MGRS and MGUS group. (**a**) The renal pathology of MGRS group. (**b**) The renal pathology of MGUS group. (*TMA* thrombotic microangiopathy, *MIDD* monoclonal immunoglobulin deposition disease, *PGNMID* Proliferative glomerulonephritis with monoclonal immunoglobulin deposits, *LCPT* light-chain proximal tubulopathy).

**Figure 3:**
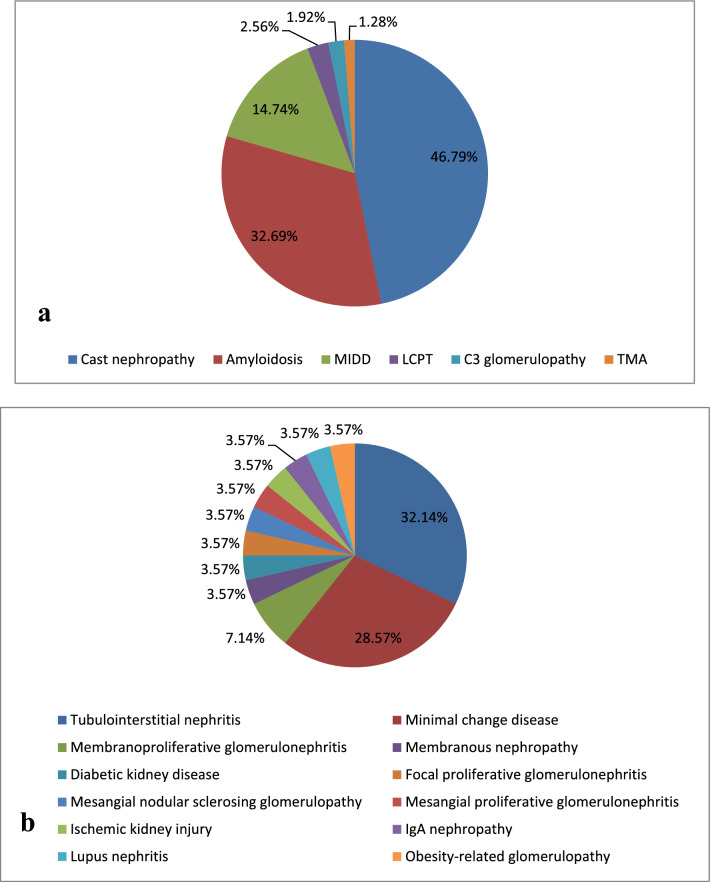
The renal pathology of hematological malignancy group. (**a**) The renal pathology of patients with paraprotein-associated lesions in the hematological malignancy group. (**b**) The renal pathology of patients with non-paraprotein-associated lesions in the hematological malignancy group. (*MIDD* monoclonal immunoglobulin deposition disease, *LCPT* light-chain proximal tubulopathy, *TMA* thrombotic microangiopathy).

A total of 703 patients met the inclusion criteria and were classified as having MGRS (n = 260, 36.98%), MGUS (n = 259, 36.84%), or hematological malignancy (n = 184, 26.17%). Amyloidosis was the most common diagnosis of MGRS, accounting for 74.23% (n = 193), followed by TMA (n = 23, 8.85%), and monoclonal immunoglobulin deposition disease (MIDD) (n = 22, 8.46%) (Fig. 2a). Membranous nephropathy (MN) (n = 102, 39.38%) was the leading diagnosis of MGUS, followed by IgA nephropathy (n = 30, 11.58%), and diabetic nephropathy (n = 26, 10.04%) (Fig. 2b). The renal pathological changes of patients with hematological malignancies were divided into two categories: (1) paraprotein-associated lesions (n = 156, 84.78%), where cast nephropathy (n = 73, 46.79%) was the leading diagnosis, followed by amyloidosis (n = 51, 32.69%), and MIDD (n = 23, 14.74%) (Fig. 3a), and (2) non-paraprotein-associated lesions (n = 28, 15.22%), where tubulointerstitial nephritis (n = 9, 32.14%) was the leading pathological finding, followed by minimal change disease (n = 8, 28.57%) and membranoproliferative glomerulonephritis (n = 2, 7.14%) (Fig. 3b). The other lesions are summarized in Figs. 2 and 3.

### Clinical and hematological characteristics of patients with MG and renal damage

The clinical characteristics of patients with MG and renal damage are summarized in Table 1. Patients with MGRS had a lower incidence of hypertension than those with MGUS or hematological malignancies. Patients with MGRS had a higher mean serum creatinine level than patients with MGUS, but a lower serum creatinine level than those with hematological malignancies. The mean serum C3 level was lower in patients with hematological malignancies than in those with MGRS or MGUS. Patients with MGRS had a higher median 24-h urine protein excretion than those with MGUS or hematological malignancies. Nephrotic syndrome was diagnosed in 70.4%, 45.2%, and 27.2% of patients with MGRS, MGUS, and hematological malignancies, respectively. Patients with MGUS were more likely to have hematuria than those with MGRS or hematological malignancies. Patients with hematological malignancies had a higher mean plasma cell percentage in the bone marrow on renal biopsy than those with MGRS or MGUS. Gender and age were not statistically different among patients with MGRS, MGUS, and hematological malignancies.

**Table 1:** The clinical characteristics at renal biopsy of 703 patients with MG and renal damage.

Characteristics	MGRS lesions n = 260	MGUS lesions n = 259	hematological malignancy lesions n = 184	*P*-value
Male, n (%)	155 (59.6)	166 (64.1)	111 (60.3)	0.54
Age, year	60 (52, 66)	57 (48, 65)	57 ± 10.46	0.06
Hypertension, n (%)	108 (41.5)	151 (58.3)	81 (44)	< 0.001
Nephrotic syndrome, n (%)	183 (70.4)	117 (45.2)	50 (27.2)	< 0.001
Renal insufficiency, n (%)	119 (45.8)	71 (27.4)	129 (70.1)	< 0.001
Serum studies				
Hemoglobin, g/L	113 (94, 130)	122.5 ± 24.1	96 (79, 111)	< 0.001
Albumin, g/L	25.2 (20.6, 30)	29.72 ± 7.79	35.5 (28.33, 41.28)	< 0.001
Creatinine, μmol/L	108 (73, 191)	81 (66, 121)	187 (86.5, 397)	< 0.001
eGFR, ml/min/1.73 m^2^	61.34 (36.08, 91.38)	77.37 (46.54, 97.16)	40.1 (25.1, 64.3)	< 0.001
C3, g/L	1.06 (0.92, 1.33)	1.12 (0.9, 1.31)	1.04 ± 0.32	0.024
C4, g/L	0.3 (0.23, 0.36)	0.27 (0.2, 0.34)	0.3 (0.24, 0.39)	0.001
Urinary studies				
Urinary protein, g/d	4.8 (2.74, 7.12)	3.47 (1.29, 6.61)	3.36 (1.7, 6.32)	< 0.001
Hematuria, n (%)	82 (31.5)	148 (57.1)	56 (30.4)	< 0.001
Bone marrow biopsy				
Plasma cell percentage	2.8 (1.2, 4.8)	0.8 (0.4, 2.4)	18 (9.2, 33.8)	< 0.001

Values for continuous variables are described as mean ± SD or median (IQR) depending on the distribution, and categoric variables are described as count (%).

In the multivariate logistic regression model that considered gender, age, diagnosis of hypertension, proteinuria ≥ 1.5 g/d, C3 hypocomplementemia, abnormal FLC ratio, hematuria, renal insufficiency, and nephrotic syndrome, the clinical indicators related to the MGRS were the presence of nephrotic syndrome (OR, 3.406; 95% CI, 1.828 to 6.345; *P* < 0.001) and an abnormal FLC ratio (OR, 1.792; 95% CI, 1.003 to 3.202; *P* = 0.049). (Table 2).

**Table 2:** Clinical characteristics associated with a diagnosis of MGRS in patients with MG and renal damage.

Indicators	Odds ratio (95% confidence interval)	*P* Value
Male	0.514 (0.304, 0.871)	0.013
Age	1.021 (0.997, 1.045)	0.086
Hypertension	0.835 (0.501, 1.391)	0.488
Proteinuria ≥ 1.5 g/d	0.738 (0.343, 1.587)	0.437
C3 hypocomplementemia	1.192 (0.618, 2.297)	0.600
Abnormal FLC ratio	1.792 (1.003, 3.202)	0.049
Hematuria	0.554 (0.318, 0.965)	0.037
Renal insufficiency	1.225 (0.713, 2.105)	0.462
Nephrotic syndrome	3.406 (1.828, 6.345)	< 0.001

The hematological characteristics of patients with MG and renal damage are summarized in Table 3. The abnormal rate of the serum FLC ratio and positive rates of SPE, serum IFE, and urine BJP electrophoresis were higher in patients with hematological malignancies than in those with MGRS or MGUS.

**Table 3:** The hematological characteristics of 703 patients with MG and renal damage.

	MGRS lesions, n = 260	MGUS lesions, n = 259	hematological malignancy lesions, n = 184	*P*-value
SPE positive, n (%)	164/222 (73.87)	152/220 (69.1)	131/158 (82.9)	0.009
Serum IFE positive, n (%)	199/252 (79)	177/247 (71.66)	159/175 (90.86)	< 0.001
Urine BJP electrophoresis positive, n (%)	182/246 (74)	102/243 (42)	160/183 (87.4)	< 0.001
Abnormal serum FLC ratio, n (%)	101/124 (81.45)	31/98 (31.6)	71/77 (92.2)	< 0.001

The SPE positive (36.69%), serum IFE positive (37.2%), urine BJP electrophoresis positive (40.99%), and abnormal serum FLC ratio (46.6%) had distribution peaks in the MGRS group (Supplementary Fig. S2).

### Types of MIg and subgroup distributions of patients with detectable MIg on serum IFE

Of the 703 patients with MG and renal damage, 535 had a positive result on serum IFE, among which the most common MIg was IgG λ (n = 140, 26.17%), followed by IgA λ (n = 91, 17.01%) and λ light chain (n = 87, 16.26%). IgG λ (n = 58, 29.15%) was the leading MIg in patients with MGRS, followed by λ light chain (n = 42, 21.11%) and IgA λ (n = 41, 20.6%). IgG λ (n = 48, 27.12%) was predominant in patients with MGUS, followed by IgG κ (n = 43, 24.29%) and IgA λ (n = 31, 17.51%). λ light chain (n = 40, 25.16%) was the leading MIg in patients with hematological malignancy, followed by IgG λ (n = 34, 21.38%) and κ light chain (n = 27, 16.98%). The additional MIg types are summarized in Table 4.

**Table 4:** The MIg types of patients with detectable MIg on serum IFE.

MIg types	Overall, n = 535	MGRS lesions, n = 199	MGUS lesions, n = 177	hematological malignancy lesions, n = 159
IgG λ, n (%)	140 (26.17)	58 (29.15)	48 (27.12)	34 (21.38)
IgG κ, n (%)	82 (15.33)	25 (12.56)	43 (24.29)	14 (8.81)
IgG κ and IgG λ, n (%)	1 (0.19)	0 (0)	1 (0.56)	0 (0)
IgG κ and IgM λ, n (%)	1 (0.19)	0 (0)	1 (0.56)	0 (0)
IgG λ and IgA λ, n (%)	3 (0.56)	1 (0.50)	0 (0)	2 (1.26)
IgG λ and IgA κ, n (%)	1 (0.19)	1 (0.50)	0 (0)	0 (0)
IgA λ, n (%)	91 (17.01)	41 (20.60)	31 (17.51)	19 (11.95)
IgA κ, n (%)	28 (5.23)	3 (1.51)	18 (10.17)	7 (4.40)
IgA κ and IgA λ, n (%)	1 (0.19)	1 (0.50)	0 (0)	0 (0)
IgM λ, n (%)	17 (3.18)	6 (3.02)	10 (5.65)	1 (0.63)
IgM κ, n (%)	25 (4.67)	4 (2.01)	16 (9.04)	5 (3.14)
IgM κ and IgM λ, n (%)	1 (0.19)	0 (0)	0 (0)	1 (0.63)
IgD λ, n (%)	13 (2.43)	4 (2.01)	1 (0.56)	8 (5.03)
IgD κ, n (%)	1 (0.19)	0 (0)	0 (0)	1 (0.63)
κ light chain, n (%)	43 (8.04)	13 (6.53)	3 (1.69)	27 (16.98)
λ light chain, n (%)	87 (16.26)	42 (21.11)	5 (2.82)	40 (25.16)

IgG λ (41.43%), IgA λ (45.05%), and λ light chain (48.28%) had distribution peaks in the MGRS group. IgG κ (52.44%), IgA κ (64.29%), IgM κ (64%), and IgM λ (58.82%) showed distribution peaks in the MGUS group. IgD κ (100%), IgD λ (61.54%), and κ light chain (62.79%) had distribution peaks in the hematological malignancy group (Supplementary Fig. S3).

### Study outcomes

**Figure 4:**
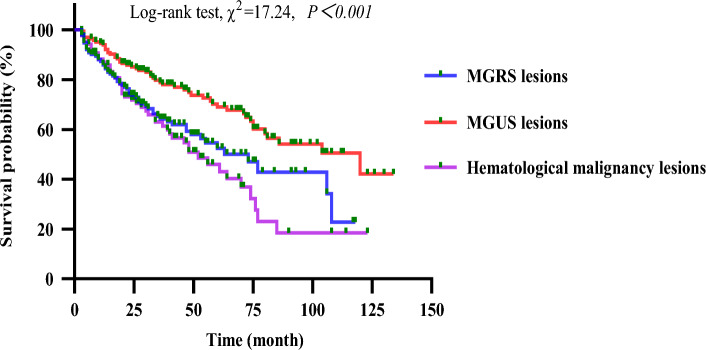
Kaplan–Meier curves of patients with MG and renal damage.

Among the 703 patients with MG and renal damage, 410 (58.32%, 410/703) were followed up for ≥ 3 months and were considered for the survival analysis. Of the 410 patients, 50 (12.20%) were lost to follow-up. During a median follow-up of 34 months (IQR, 19–58 months), renal disease progression was observed in 71 of 410 (17.32%) patients, and all-cause death occurred in 86 of 410 (20.98%) patients. Among 410 patients with MG and renal damage, the study outcomes occurred in 60 of 155 (38.71%) patients with MGRS lesions, 52 of 168 (30.95%) patients with MGUS lesions, and 45 of 87 (51.72%) patients with hematological malignancy lesions (χ^2^ = 10.484, *P* = 0.005). The Kaplan–Meier curves in Fig. 4 show that patients with MGUS lesions had better overall survival than those with MGRS or hematological malignancy lesions (*P* < 0.05), although there was no significant difference between the MGRS and hematological malignancy groups (*P* > 0.05).

## Discussion

Over the past few decades, the prevalence of MG has drastically increased, and the relationship between MG and renal damage has been increasingly recognized. MGUS, first proposed by Robert Kyle^12^, is characterized by a serum MIg concentration < 3 g/dL, a bone marrow plasma cell percentage < 10%, and the absence of end-organ damage and myeloma events. In China, the prevalence of MGUS is 0.53%, and increases with age^13^. MGRS accounts for 10% of MGUS cases and is predominant among the elderly^14^. Therefore, with the aging population in China, the incidence of both MGRS and MGUS is projected to gradually increase.

In contrast to MGUS, which does not require treatment^15^, clone-directed therapy is recommended for patients with MGRS to preserve renal function and reduce the risk of MGRS recurrence after renal transplantation^16^. Clone-directed therapy is also used in the treatment of MM, WM, and other hematologic cancers^17,18^, but has significant negative effects and is typically costly. Therefore, understanding the relationship between renal damage and MG is necessary.

In our cohort, we found that the most prevalent MGRS type was amyloidosis, and AL amyloidosis (n = 132, 68.39%) was the most prevalent amyloidosis type, accounting for more than half of all patients with MGRS, which was consistent with previous renal biopsy series^19–22^. In this study, MN was the most prevalent histopathological change in the MGUS group, similar to the findings of the Peking University First Hospital study^22^, whereas arteriosclerosis was the most common MGUS type in the Mayo Clinic study^20^, indicating that racial differences may also influence the distribution of renal diseases. Similar to previous studies^23–26^, cast nephropathy was the most common finding in the hematological malignancy group. Approximately 30–40% of patients with MM have an elevated serum creatinine level at diagnosis, and 10% present with severe renal impairment requiring dialysis^27^, showing that kidney injury is a common characteristic of MM. In our study, renal lesions related to paraprotein were encountered in 84.78% of the patients with hematological malignancy, whereas a wide range of renal disorders unrelated to paraprotein were observed in the remaining 15.22%. It is important to distinguish between these two renal lesions, because they are treated differently. Therefore, renal biopsy is essential to establish an individual diagnosis in patients with hematological malignancies.

In this study, we found that patients with MGRS had a lower incidence of hypertension and more severe proteinuria, which was consistent with previous studies^7,22,28^. Patients with MGUS were more likely to have hematuria than those with MGRS, which was similar to the finding of the Peking University First Hospital study but not consistent with the finding of the Mayo Clinic study. It was unsurprising because IgA nephropathy was one of the most common types of MGUS both in our study and in the Peking University First Hospital study, while arteriosclerosis and diabetic nephropathy were the main findings of MGUS in the Mayo Clinic study. In the MGUS group, the serum creatinine level was the lowest, which may indicate a better prognosis. In this study, patients with MGRS had a lower serum C3 level than those with MGUS, which suggested that the activation of alternative complement pathway may be associated with the occurrence of MGRS lesions. Our study showed that the presence of proteinuria ≥ 1.5 g/d was not a clinical indicator associated with MGRS lesions, which was not consistent with the findings of the Mayo Clinic and the Peking University First Hospital. In their studies, proteinuria ≥ 1.5 g/d was a risk factor associated with MGRS lesions. This difference of the result is probably due to the different inclusion criteria used, as our study did not exclude patients with hematological malignancies, while their studies did.

The IMWG guidelines recommended SPE, serum IFE, and serum FLC as initial diagnostic screening panels for patients suspected of MG^29^. In the present study, the serum IFE had a higher sensitivity than SPE in detecting the MIg, which was similar to the previous work^30^. This is related to the principles of two detection methods. SPE is traditionally carried out on agarose gel. The proteins loaded on the gel are separated into five zones based on their charge and size by electric current, albumin, α1, α2, β, and γ^31^. The MIg typically migrates in the γ-band, but it can migrate in the β-band or α-band as well. The MIg in the α-band and β-band may be unreliable to quantify due to the co-migration of physiologic proteins such as transferrin and complement proteins^32^. The electrophoretic migration of MIg may reduce the sensitivity of SPE in detecting MG. The MIg isotype can be identified by serum IFE, and the detection sensitivity of MIg can be raised by serum IFE as well^31^. The serum FLC assay can measure both isotypes of light chain, κ and λ, and it is highly sensitive and can detect both isotypes to levels that are below the normal physiological range^33^. Mass spectrometry-based techniques for clinical and analytical applications have been emerging in recent years^34^.

Consistent with previous studies^35–37^, we found that the most common heavy chain type of MIg was IgG in patients with MGRS and MGUS. The site and pathology of kidney injury are specific to the physiochemical properties rather than the amount and production rate of MIg^38^. MIg type is correlated with clinical and renal pathological features in patients with MGRS^35^. In addition, the type of MIg is an important predictor associated with the progression of MGUS to lymphoma or MM. Patients with IgM-MGUS were more likely to develop lymphoma or a related lymphoid disease, whereas those with IgG or IgA-MGUS were more likely to develop MM or a related condition^39^. Furthermore, the MIg type can be used to classify MM. Therefore, the type of MIg combined with clinical features and renal pathological changes may facilitate the individual diagnosis and treatment of patients with MG and renal damage.

This study has some limitations. First, it is regrettable that treatment interventions were not included, because many patients were lost to follow-up due to abandonment of treatment. Moreover, the retrospective nature of our cohort made it impossible to address the inherent underlying selection bias.

In conclusion, the most common pathological finding in the MGRS group was amyloidosis, and MN was the leading cause of MGUS. In the hematological malignancy group, the most prevalent paraprotein-associated disease was cast nephropathy, whereas the most common non-paraprotein-associated disease was tubulointerstitial nephritis. The presence of nephrotic syndrome and an abnormal FLC ratio were linked to MGRS. The overall survival was better in patients with MGUS than in those with MGRS or hematological malignancies. Together, our study findings describe the clinicopathological characteristics and prognosis of patients with MG and renal damage in Central China and provide a valuable reference for better understanding and diagnosis of the disease.

### Supplementary Information

Supplementary Figures.

## Data Availability

The datasets used and/or analysed during the current study available from the corresponding author on reasonable request.
