# Supplementary material for: Clinicopathological characteristics and prognosis in patients with monoclonal gammopathy and renal damage in central China: a multicenter retrospective cohort study
Source: Sci Rep. 2024 Apr 1;14:7667. doi: 10.1038/s41598-024-58467-z (PMC10984969; doi:10.1038/s41598-024-58467-z)
Supplement: Supplementary file 1 — Supplementary Figures. [file 41598_2024_58467_MOESM1_ESM.pdf]

Supplement (Supplementary Figure S1, Supplementary Figure S2, Supplementary Figure S3)

Clinicopathological characteristics and prognosis in patients with monoclonal gammopathy and renal damage in central China: a multicenter retrospective cohort study

Huimin He[1], Zheng Wang[1], Jiayun Xu[2], Yun Liu[3], Yeqing Shao[2], Yulong Hou[3], Jinping Gu[1], Ruimin Hu[1*] and Guolan Xing[1*]

[*] Corresponding authors; email: huruimin1026@126.com; xgl@zzu.edu.cn

[1]Department of Nephrology, The First Affiliated Hospital of Zhengzhou University, Zhengzhou, Henan, China.

[2] Department of Nephrology, Henan University of Science and Technology Affiliated First Hospital, Luoyang, Henan, China.

[3] Department of Nephrology, Xinxiang Medical University First Affiliated Hospital, Xinxiang, Henan, China.

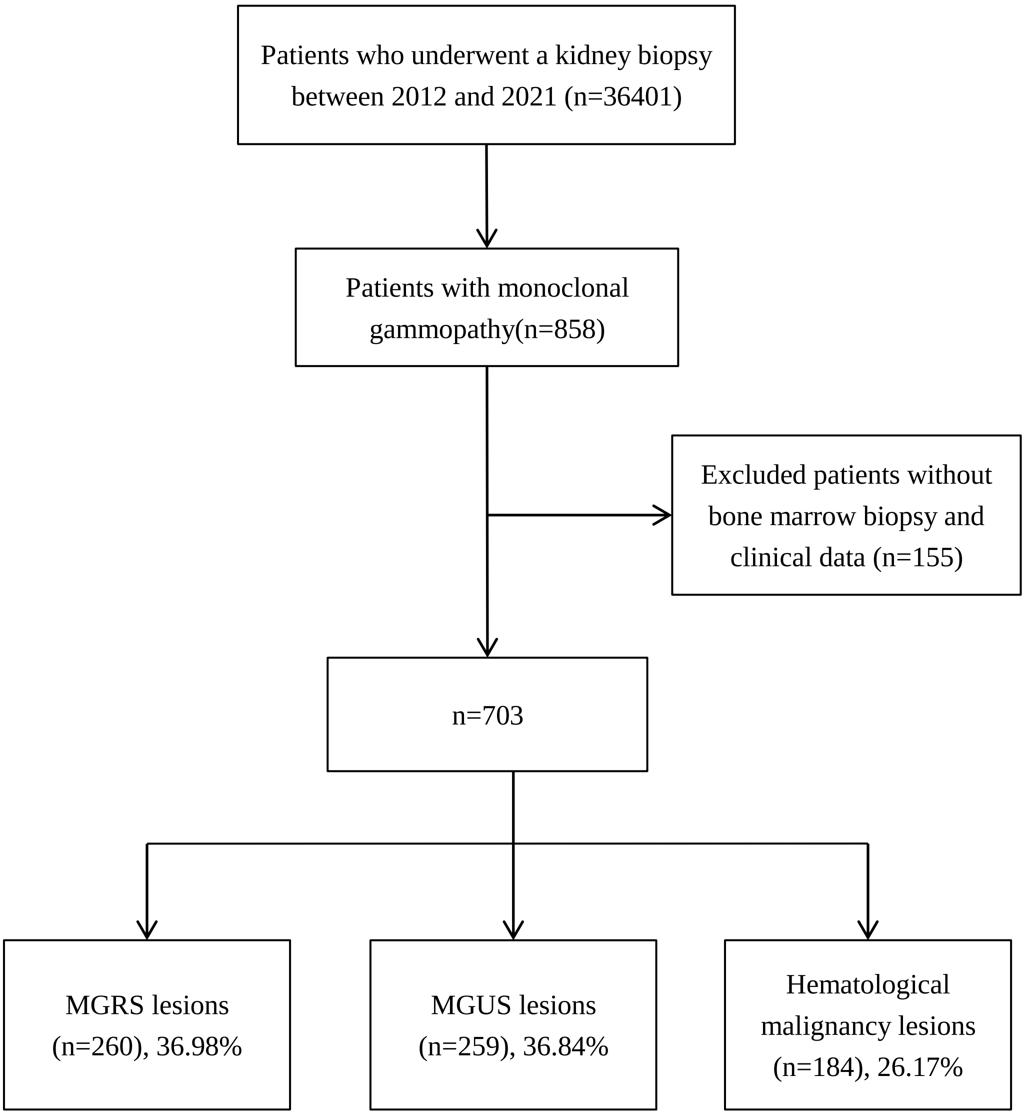

Supplementary Figure S1. Study flow chart of patients with monoclonal gammopathy and kidney injury from 2012 to 2021. (*MGRS* monoclonal gammopathy of renal significance, *MGUS* monoclonal gammopathy of undetermined significance)

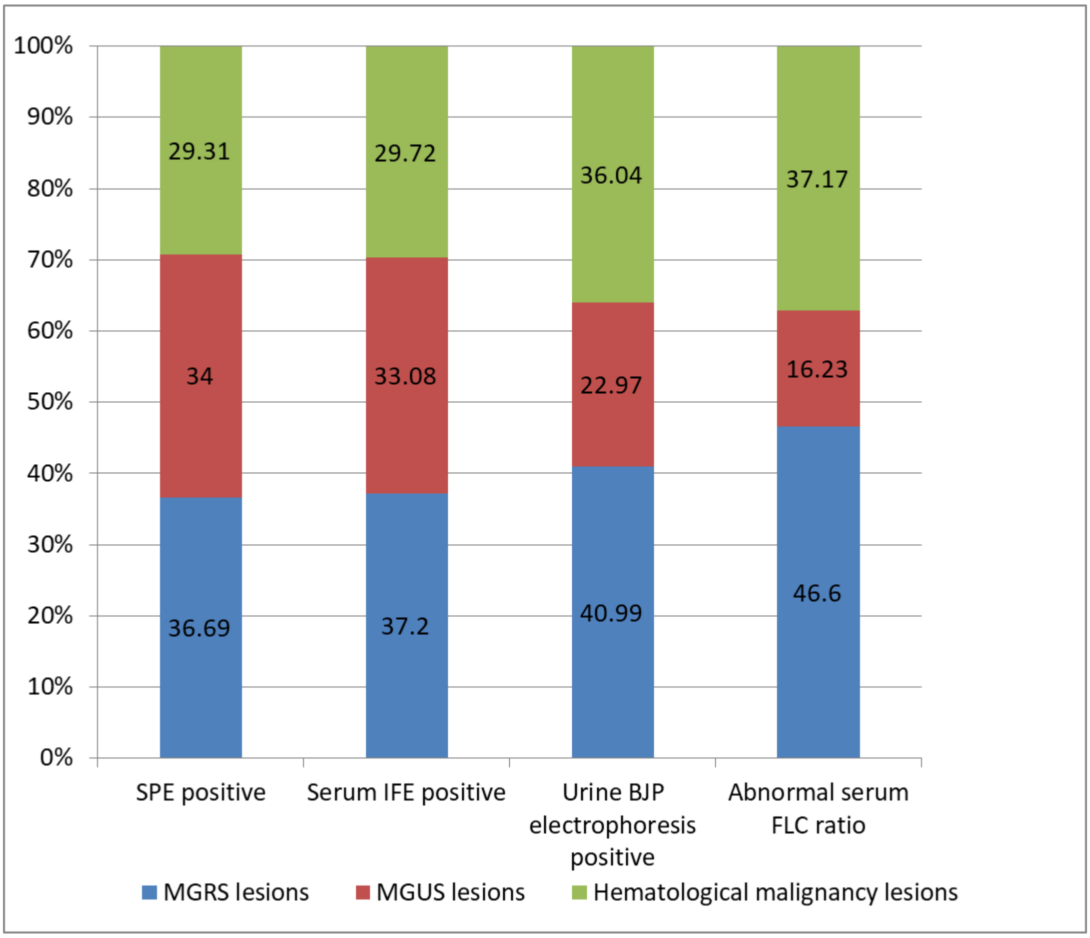

Supplementary Figure S2. The subgroup distributions according to MIg-related hematological detection methods.

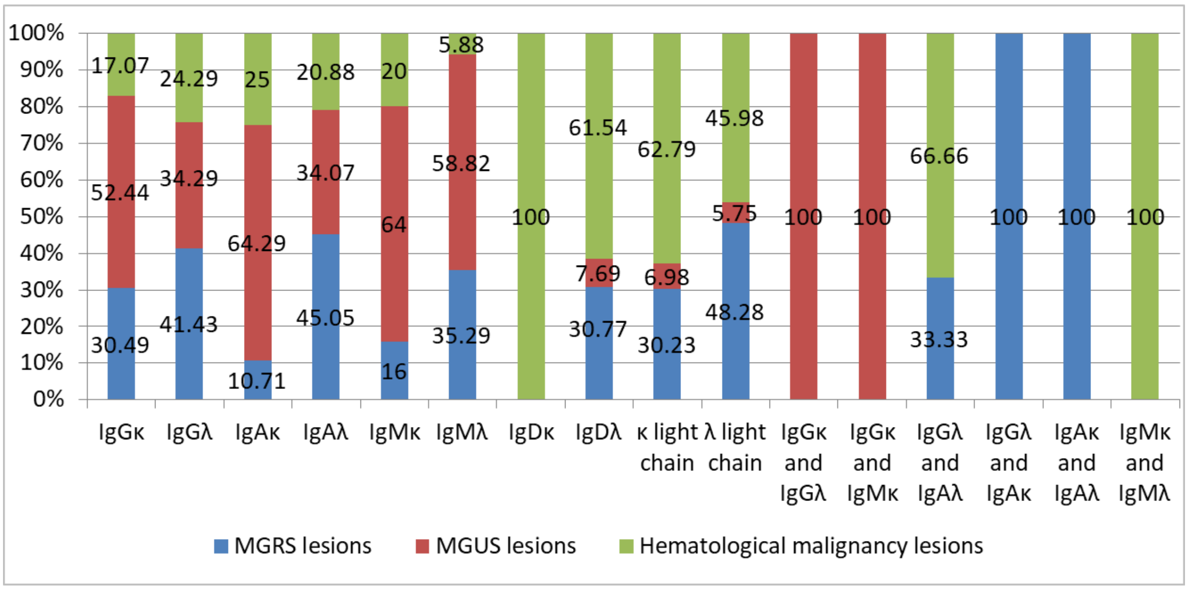

Supplementary Figure S3. The subgroup distributions according to MIg types.
